# Higher recall in metagenomic sequence classification exploiting overlapping reads

**DOI:** 10.1186/s12864-017-4273-6

**Published:** 2017-12-06

**Authors:** Samuele Girotto, Matteo Comin, Cinzia Pizzi

**Affiliations:** Department of Information Engineering, University of Padova, via Gradenigo 6/A, Padova, 35131 Italy

**Keywords:** Metagenomics reads classification, Boosting by overlapping reads

## Abstract

**Background:**

In recent years several different fields, such as ecology, medicine and microbiology, have experienced an unprecedented development due to the possibility of direct sequencing of microbioimic samples. Among problems that researchers in the field have to deal with, taxonomic classification of metagenomic reads is one of the most challenging. State of the art methods classify single reads with almost 100% precision. However, very often, the performance in terms of recall falls at about 50%. As a consequence, state-of-the-art methods are indeed capable of correctly classify only half of the reads in the sample. How to achieve better performances in terms of overall quality of classification remains a largely unsolved problem.

**Results:**

In this paper we propose a method for metagenomics CLassification Improvement with Overlapping Reads (CLIOR), that exploits the information carried by the overlapping reads graph of the input read dataset to improve recall, f-measure, and the estimated abundance of species. In this work, we applied CLIOR on top of the classification produced by the classifier Clark-l. Experiments on simulated and synthetic metagenomes show that CLIOR can lead to substantial improvement of the recall rate, sometimes doubling it. On average, on simulated datasets, the increase of recall is paired with an higher precision too, while on synthetic datasets it comes at expenses of a small loss of precision. On experiments on real metagenomes CLIOR is able to assign many more reads while keeping the abundance ratios in line with previous studies.

**Conclusions:**

Our results showed that with CLIOR is possible to boost the recall of a state-of-the-art metagenomic classifier by inferring and/or correcting the assignment of reads with missing or erroneous labeling. CLIOR is not restricted to the reads classification algorithm used in our experiments, but it may be applied to other methods too. Finally, CLIOR does not need large computational resources, and it can be run on a laptop.

**Electronic supplementary material:**

The online version of this article (doi:10.1186/s12864-017-4273-6) contains supplementary material, which is available to authorized users.

## Background

Metagenomics is the study of genomic sequences obtained from an environment such as, for example, water, saliva, soil, etc. [1]. A metagenomic sample is processed by extracting and studying the genetic material in order to detect the microorganisms that are present in each sample. Metagenomics is transforming ecology, medicine, microbiology, and other research areas investigating various microbiomes [2, 3], by enabling for the first time the genomic study of environmental samples, given the difficulty or impossibility to culture most bacteria. For example, the diversity of microbes in humans is found to be associated with diseases such as inflammatory bowel disease (IBD) [2] and colorectal cancer [3]. Metagenomics allows the identification and characterization of bacterial and viral genomes at a level of detail not previously possible. Moreover it can be used to detect previously unknown species.

Taxonomic classification of metagenomics reads can help the identification of functional potential of the microbes. In general, there are two methods to detect the taxonomic content of environmental samples: (1) sequencing phylogenetic marker genes, e.g. 16S rRNA, that requires PCR amplicons analysis; (2) Next Generation Sequencing, where all the genomic material of the sample is sequenced.

The assignment of the correct taxa using marker genes is a relative simple step. However, this method suffers the drawback of potentially biased amplification steps. Furthermore, not all taxa can be captured by traditional 16S sequencing, because of its divergent gene sequences [4].

With the continuing decrease in cost of sequencing, approaches based on Next-Generation Sequencing are becoming the most common ones. The advantages include the ability to gain insights of the genomic content of a microbiome through functional classification, without the need to culture bacteria or of biased preprocessing steps. However, the short length of NGS reads poses a number challenges for the correct taxonomical classification of each read.

Many computational methods have been developed to classify metagenomic reads. These methods can be broadly divided into three categories, reflecting their different strategies: (1) sequence similarity based methods, like MegaBlast [5] and Megan [6], in which reads are searched for in reference databases through sequence similarity; (2) marker-based methods, like [7, 8], where certain specific marker sequences are used to identify the species. Some of these methods are based on universal genes, others, like MetaPhlAn [9], utilizes marker genes that are clade specific; (3) sequence composition based methods, which are based on characteristics of their nucleotide composition (e.g. *k*-mers presence).

The fastest approaches are sequence composition based methods. They generate models from the reference organisms genomes, usually based on *k*-mers counts. Then the input reads are classified based on which model fit. In this category the most representative methods are Kraken [10], Clark [11] and Lmat [12], in which reads are queried against reference databases and the origin of the hit sequences is used to classify reads. These methods are able to achieve a precision in the classification task as good as MegaBlast, but they are much faster and they can handle larger datasets.

All these tools focus on the improvement of the correct assignment of reads to the taxa they belong in terms of precision. Indeed they have reached very high levels, e.g. Clark [11] reports precisions above 95% on many datasets. On the other hand, in terms of recall, i.e. the percentage of reads actually classified, both Clark and Kraken usually show performances between 50% and 60%, and sometimes on real metagenomes just 20% of reads can be assigned to some taxa.

The literature in this field is growing very rapidly (see, for example, [6, 7, 9–14]) with a focus on improving the classification precision. Instead, in this study we aim to improve the recall in order to reduce the number of unclassified reads, that is one of the major issues when analyzing real microbiome datasets.

Recently, we have develop MetaProb [14] a method for binning metagenomic reads. MetaProb is able to effectively group reads together to form bins of reads that represent candidate species. As several other binning tools like MetaCluster [15] and BiMeta [16], MetaProb is based on the construction of the reads overlap graph [17] to mimic the assembly process. However, MetaProb does not rely on a reference database and it cannot taxonomically annotate reads, or bins. In this paper we introduce a novel boosting method for CLassification Improvement with Overlapping Reads (CLIOR), that relies on the definition of overlapping reads graph, and it uses as input the taxonomic label assignments given by Clark, a standard single reads classifier. The problem with many single reads classifiers is that reads that share a considerable overlap might be assigned to different taxa. Our method considers groups of overlapping reads as originating from a same species, thus all the reads that belong to a given group will be labeled with the same taxa, leading to an improvement of classification performances.

The proposed CLIOR approach is characterized by four distinctive features: i) a pipeline that combines reads overlaps together with theirs taxonomic labels; ii) the introduction of a classification correction, that will reduce the problem of unassigned reads, and correct misclassified reads; iii) CLIOR does not depend on a specific algorithm for the classification of single reads, e.g. we used Clark, but it can applied to other methods too; iv) the ability of CLIOR to improve reads annotations increases as the size of datasets grows.

## Methods

**Fig. 1:**
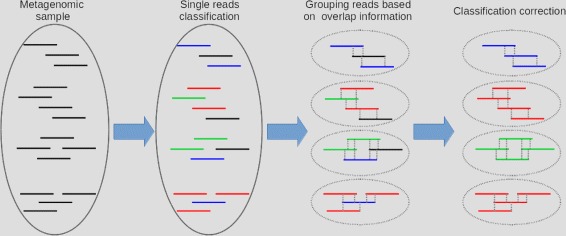
CLIOR pipeline. The tree main phases of CLIOR: classification; grouping; voting. In black the reads that are not assigned; in blue the reads assigned to species A’ in red those assigned to species B, and in green those assigned to species C

In this section we will describe the CLIOR approach to improve the overall quality of classification of metagenomic samples. With reference to Fig. 1 we will describe the main steps of our approach, namely: i) initial classification of the input read datasets with a state-of-the-art classifier; ii) partitioning of the reads into homogeneous groups; iii) adopt a voting strategy and apply it to each group to obtain the final classification.

### Initial classification of reads

The set of reads obtained from a metagenomic sample is given in input to a classifier that will try to assign a label to each read.

After this step a read is assigned a label at the taxonomy level for which the software is run if, according to the classifier-specific strategies, the confidence level of the call is above a given threshold. Otherwise the read remains unlabeled. The comparison of different metagenomic classification methods is a non-trival problem and it is not the scope this paper. We chose Clark [11] as one of the most promising tools in terms of both similarity to the correct answer and the fraction of reads classified [18]. However, CLIOR is of general use and it can take in input the taxonomic annotations produced by any other tool.

The next steps in our approach have the objective of both reducing the cardinality of the set of unlabeled reads, and correcting labels that could have been erroneously assigned to some reads.

### Grouping reads based on overlaps

In this step of our pipeline, the set of reads is partitioned into groups, based on the extent of their overlap. Ideally the overlap would be computed using a reads overlap graph [17], but this approach is highly demanding in terms of RAM. Instead we use an alignment-free technique used in de-novo genome assembly, and that has been also successfully applied as an intermediate step in several metagenomic binning studies [14–16, 19].

The overlap between reads is estimated by considering the amount of shared *k*-mers between reads. This technique relies on the assumption that, by choosing a sufficiently large value for *k*, the probability that two *k*-mers are shared by different genomes is low. For example, a study presented in [16] shows that the average ratio of common *k*-mers between pairs of bacterial genomes is less than 1.02% when *k*=30. Therefore, the presence of a shared *k*-mer between two reads should indicate that the two reads belong to the same species. Moreover, if several such *k*-mers are shared, they are also actually likely to be overlapping reads. This strengthens the probability to meet our goal of having in the same group only reads from the same species.

The partitioning of the set of reads into groups is obtained as follows. The input reads are scanned one at the time, and a global inverted index is built to store for each *k*-mer the list of reads in which it occurs. Then an approximate reads overlap graph is built, in which a node represents a read, and an edge is inserted between two reads *if and only if* the dot product of their *k*-mer vectors is greater than a pre-defined threshold *m*. In fact, when analyzing datasets with millions of reads, it is crucial to store only qualifying edges, rather than the full graph, in order to limit the RAM usage. This approach allows us to analyze datasets an order of magnitude larger than the full graph approach.

Given a read *R*_*i*_, its composition vector is a vector *V*_*i*_ of size 4^*k*^ where the entry *V*_*i*_[*t*] holds the number of occurrences of the *t*-th *k*-mer in lexicographic order.

In order to compute such a dot product without actually build the 4^*k*^ sized vectors, we proceed as follows. Consider a read *R*_*i*_ and its set of *k*-mers $K_{R_{i}}$. For each $w \in K_{R_{i}}$ we query the inverted index to get the corresponding list *L*_*w*_ of reads that share the *k*-mer *w*. For each read *R*_*j*_∈*L*_*w*_, we count its multiplicity in the list and multiply this value by the multiplicity of *w* in $K_{R_{i}}$. This value is kept in a variable *W*_*i*,*j*_ that stores the partial weight of the edge between *R*_*i*_ and *R*_*j*_. We then proceed with the next *k*-mer in *R*_*i*_ and if *R*_*j*_ is found again in the corresponding list, then the result of the multiplication is added to *W*_*i*,*j*_. Note that, while processing read *R*_*i*_, we do not need to keep all the variables *W*_*i*,*j*_ in memory for all *j*, because only few reads *R*_*j*_ will share some *k*-mers. Since *W*_*i*,*j*_ is a sparse vector, we use an unordered map to store only the required fields. When all the *k*-mers of *R*_*i*_ have been considered, we check the computed weights and insert an edge between *R*_*i*_ and *R*_*j*_ if and only if *W*_*i*,*j*_≥*m*, for a given threshold *m*. We then proceed with the next read to be processed. The values of the read length (about 100 bases) and of the *k*-mer length (*k*=30) are usually such that the dot product we compute often coincides with the number of shared *k*-mers between two reads. In fact its very unlikely that a short read contains the same 30-mer twice.

Once the overlap graph has been set up, the grouping phase begins. Initially, each read is considered as a group, and groups are progressively merged until a stopping criteria is met. Starting from a node, we explore its outgoing edges, and include in its group the node among its neighbors that shares with the group the highest number of edges. Then the new frontier is considered and we iterate the process until the size of the group has reached a threshold *T*. The role of this threshold is to avoid that, by growing too large, the groups might end up including reads from two different species linked by few, but sufficient, common reads. However, when computing the size of a group, it is not enough to sum the lengths of its reads, because overlaps between reads should not be counted more than once. To avoid overcounting these overlaps, while building a group we also select a subset of independent reads within the group. An independent set, defined on a graph, is a set of vertices which does not contain adjacent vertices, thus they do not overlap. Since the problem of finding the maximum independent set *I*(*G*) of a group *G* is known to be NP-hard, we adopt a greedy solution. If the read chosen for extension is not adjacent to any read in *I*(*G*), then this is a new independent read and we add *x* to *I*(*G*). The stopping criteria is met when the length of the reads in *I*(*G*), which by definition do not overlap, reaches a predefined value *T*.

When we cannot add further nodes, the group is defined, and all the nodes in it are marked as visited. The process is repeated starting from an unvisited node until the whole graph has been explored.

### Voting groups of reads

The method described above, with conservative parameters *k*=30 and *T*=9000 (see “Parameters and evaluation metrics” subsection), is able to cluster reads into several small groups. Note that each species is not completely contained in a group, but in general it is scattered across different groups. With reference to Fig. 1, this situation is represented by the species labeled in red. However, the groups are characterized by a very high precision, in fact on simulated datasets about 99.9% of groups contains reads from one species. Therefore, we expect the reads within a group to be assigned a same label. However, most classifiers ([10, 11]) leave many reads unassigned. Here is where our approach can be successfully exploited. When inspecting the label assignment within a group, if there are unassigned reads, or if several different labels are present, we proceed with a re-assignment following a majority consensus vote. Experiments where we do not re-assign labels if the reads have already been classified by Clark showed similar, but slightly lower, performances than the ones we obtained with re-assignment (see Additional file 1).

In case of ties, we proceed by randomly picking a winner label and assigning that to all the reads in the group. While this solution could potentially end up in the re-assignment of reads that were initially correctly classified, we argue that the impact in such a case is indeed limited. In fact, in order to have a relevant impact we should have ties in big groups. However, exact ties in big groups are quite unlikely to happen, because of the high precision of groups. For small groups we have an higher probability, but the effect of wrong re-labeling is also limited to a small number of reads, thus not affecting substantially the final results. In terms of numbers over all experiments, at species level, 1.8% of groups have ties. The percentage of reads whose labeling is affected by the random choice of the winner is about 0.6%. At genus level, 0.61% of groups have ties, and only than 0.13% of the reads are affected.

## Results and discussion

We used several metagenomic datasets to test the performance of CLIOR on different simulated and real communities. The simulated and real datasets used come from other papers ([10, 11, 16]) and we summarize them in the next section.

### Simulated and synthetic metagenomes

For our test we used 16 sets of *simulated* short read datasets used also in [16]. These are generated with MetaSim software and they can be partitioned in two groups: S and L. Each dataset comprises paired-end short reads (length of approximately 80 bp) generated according to the Illumina error profile with an error rate of 1%. The six datasets in L are built over the genomes of two species, *Eubacterium eligens* and *Lactobacillus amylovorus*, but they are characterized by a different abundance ratio between the two species. The ten datasets in S are much more varied in terms of number of species (up to 30), abundance ratio (balanced/unbalanced), and phylogenetic distance (details on [16]).

We included in our tests also five mock communities (*synthetic* datasets) that are constructed from real sequencing data, called: MiSeq, HiSeq, MK_a1 and MK_a2, simBA5. The MiSeq and HiSeq metagenomes were built using 10 sets of bacterial whole-genome shotgun reads, as in Kraken [10]. We use the dataset of short-reads Illumina HiSeq, to create the MK_a1 and MK_a2 datasets with two abundance profiles.

The datasets HiSeq and MiSeq contain 10000000 and 4000000 single-end reads respectively, MK_a1 and MK_a2 have 1000000 paired-end reads for a total 2000000 reads to classify. The MiSeq dataset is particularly difficult to analyze because it contains five genomes from the *Enterobacteriaceae* family (C*itrobacter, Enterobacter, Klebsiella, Proteus and Salmonella*). This can make the classification step of these taxa more difficult because these species have an high sequence similarity [10].

As described in [10] simBA5 metagenome was created by simulating reads from the complete set of bacterial and archaeal genomes in RefSeq, for a total of 1216 species. It was created with an high error rate, to evaluate the performance on data that contain many errors or have strong differences from the genomic library available (for more details see [10]).

Since our algorithm used Clark, we needed to compute the reference database based on the NCBI RefSeq. However, this collection is not complete and we have some reads which cannot be correctly identified. Examples are the reads in HiSeq metagenomes with the *Pelosinus fermentans* species and in MiSeq metagenome with *Proteus vulgaris*, that are incorrectly classified for the same reason [11].

### Real data

To evaluate the performance of our algorithm we also analyzed five real experiments taken from NCBI and that were also used for the evaluation of Clark and Kraken. We created all datasets filtering human reads and sampling in a uniform manner so as not to change the characteristics of the datasets. As in [11] we used SRS015072 (mid-vagina, two runs, SRR062276 with 698428 reads and SRR062301 with 692906 reads), SRS019120 (saliva, two runs, SRR062415 and SRR062462 both with 2000000 reads) and SRS023847 (anterior nares, one run, SRR061942 with 600000 reads).

### Parameters and evaluation metrics

In this paper we used CLIOR to boost the performance of Clark-l, so we need to set the parameters for both. Among the available versions of Clark, we used Clark-l, the *light* version of Clark, with all default parameters. This choice is motivated by our objective to obtain performances comparable or better than the state-of-the art algorithms, but using much less computational resources. In fact Clark-l can be run on a laptop. As for CLIOR, we set the threshold *m* (minimum threshold of shared *k*-mer, *k*=30) in different ways on the basis of the properties of datasets. Several previous work (e.g. [14, 16]) showed that a good value for *m*, for short reads, is *m*=5. Ideally we would have run all our experiments with this value. However, HiSeq and MiSeq are big datasets (10M and 4M reads, respectively). In order to be able to analyze these datasets we had to increase the threshold to *m*. In fact, smaller values of *m* correspond to denser graphs, thus increasing the memory requirements. The choice of *m* reflects a trade-off between precision and available computational resources. In our experiments the parameter *m* of CLIOR was set to 45 for the HiSeq and MiSeq datasets, and *m*=5 for all other tests. The parameter *T* of CLIOR was set to 9000, similarly to other studies [14, 16].

In order to compare the results of CLIOR with Clark-l, we used precision, recall and F-measure metrics with definitions as in [11]. Given *N* the number of reads, *Y* the number of reads classified and *X* the number of correctly classified reads, we can define precision as *P*=*X*/*Y*, that is the fraction of correct assignments over the total number of assignments, and recall as *R*=*X*/*N*, that is the ratio between the number of correct assignments and the number of reads to be classified. The F-measure emphasizes comprehensively on both precision and recall, being defined as *F*=2*P* *R*/(*P*+*R*). The aim of this study is to improve the classification quality, in particular by increasing the recall without affecting the performances of the others metrics.

In our algorithm the processing of reads, to create the overlap graph, is the most demanding phase and it requires RAM to store the *k*-mers and the graph edges. With our setup we were able to run CLIOR on all tests in a PC with a Intel core i7-4510U CPU @ 2.00 GHz x 4 with 16 GB of RAM and 10 GB of swap partition, with a maximum amount of memory needed of 24 GB.

### Results on simulated and synthetic metagenomes

**Fig. 2:**
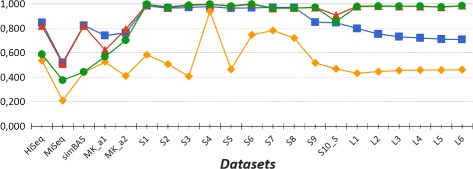
Precision and recall at species level. Comparison between Clark-l and CLIOR precision and recall on simulated and synthetic datasets. In blue Clark-l precision, in yellow Clark-l recall. In red CLIOR precision, in green CLIOR recall

**Fig. 3:**
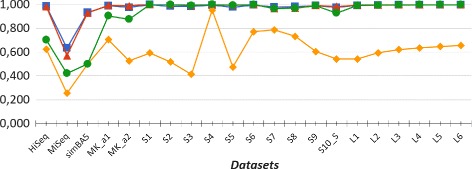
Precision and recall at genus level. Comparison between Clark-l and CLIOR precision and recall on simulated and synthetic datasets. In blue Clark-l precision, in yellow Clark-l recall. In red CLIOR precision, in green CLIOR recall

**Fig. 4:**
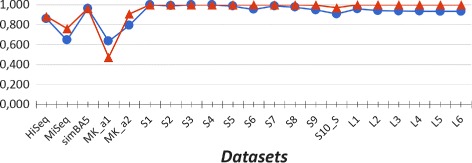
Pearson correlation at species level. Comparison between Clark-l and CLIOR on simulated and synthetic datasets. In blue Clark-l, in red CLIOR

**Fig. 5:**
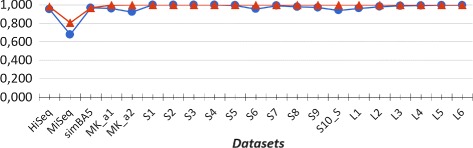
Pearson correlation at genus level. Comparison between Clark-l and CLIOR on simulated and synthetic datasets. In blue Clark-l, in red CLIOR

In our experiments we evaluated the performances of CLIOR and Clark-l at both species and genus level of classification. We report in Table 1 a summary of the resulting average precision, recall and f-measure on both simulated and synthetic metagenomes at both species and genus level. A detailed comparison on each dataset is shown in Fig. 2 for the analysis at species level, and in Fig. 3 for the analysis at genus level. Note that in Figs. 2, 3, 4, and 5 adjacent points are not related, therefore the role of the lines between them is purely that of enhancing the visualization. The corresponding full tables are shown in the Additional file 2.

**Table 1:** Genus-level and species-level average accuracy of CLIOR and Clark-l, for various simulated (*N*=16) and synthetic (*N*=5) metagenomes

Dataset	Tool	Species			Genus		
		Prec	Rec	F-m	Prec	Rec	F-m
**Simulated**	Clark-l	0.869	0.555	0.670	0.988	0.629	0.762
	CLIOR	0.978	0.973	0.976	0.992	0.986	0.989
	Diff	**0.109**	**0.419**	**0.306**	**0.004**	**0.357**	**0.227**
**Synthetic**	Clark-l	0.740	0.425	0.537	0.902	0.522	0.656
	CLIOR	0.714	0.536	0.607	0.892	0.680	0.765
	Diff	**-0.026**	**0.110**	**0.070**	**-0.010**	**0.159**	**0.109**

Diff is the difference between the corresponding CLIOR and Clark-l performances

First of all, if we compare the overall results obtained at species and genus level, we can see that, for both CLIOR and Clark-l, the performances at genus level are better than those at species level. This was indeed expected. In fact, in the taxonomy tree, when the classification level is more specific, the label assignment is more difficult. Moreover, it is possible that, although at species level a read is assigned a wrong label, at genus level the same label is indeed correct, thus making genus level classification relatively less difficult.

By observing more in detail the performances of CLIOR, we can see that there is a generalized improvement in terms of recall. An important aspect is that this does not come at great expense of precision. On the contrary, on average, among the simulated datasets we have an improvement also for what concern the precision. In the simulated dataset the gain in precision is on average 10% and 0.4%, for the two different levels of classification.

For what concerns the recall, the average increments are 41.86% and 35.72% for simulated datasets, at species and genus level, and 11.03% and 15.88% for synthetic datasets at the same two levels. The major recall increment is on the dataset S3, both for species (about 57%) and for genus level accuracy (about 58%).

One of the most difficult datasets is the synthetic metagenome MiSeq, that contains five genomes from the same family. Five species of the same family and high sequence similarity may influence the performances of Clark-l. In fact, for the MiSeq metagenome, Clark-l classified correctly only 21% and 25.56% of reads at the species and genus level. Moreover, its precision is the lowest among all the datasets that we analyzed, specifically 52% and 63%. On this difficult dataset CLIOR is still able to improve the recall by 16% and 17%, whereas the precision decreases by 1% and 6% for different target levels.

When analyzing precision and recall values, it is of interest to have also the actual number of reads correctly assigned, in order to have a complete picture. We report in Additional file 3 the number of reads correctly classified and the number of assignments for species and genus level classification. These absolute numbers show that for the MiSeq dataset, although we have a moderate loss of precision, still the number of reads correctly classified grows from 5369370 (Clark-l) to 5871490 (CLIOR) at the species-level and from 6242990 (Clark-l) to 7022260 (CLIOR) at genus-level. Similar observations hold also for the datasets MK_a1, that is the one with the highest drop of precision, we have that Clark-l classify correctly 1051590 reads at species-level, whereas CLIOR 1135770 reads; at the genus-level we have 1406490 (Clark-l) and 1806830 (CLIOR) correct reads. Note that the number of reads correctly assigned to a species plays a crucial role for the robust identification of the species in a sample.

### Estimating the species abundance

An interesting question is to which extent the abundance of a given species (or genus), predicted by our method CLIOR, resembles the actual abundance of that species (or genus) in the input dataset. To answer this question, in line with other studies [18], we compute the Pearson correlation coefficient between abundance, predicted by CLIOR and Clark-l, and the actual composition of species and genera in the datasets. The results are shown in Figs. 4 and 5 for species and genus analysis respectively.

**Fig. 6:**
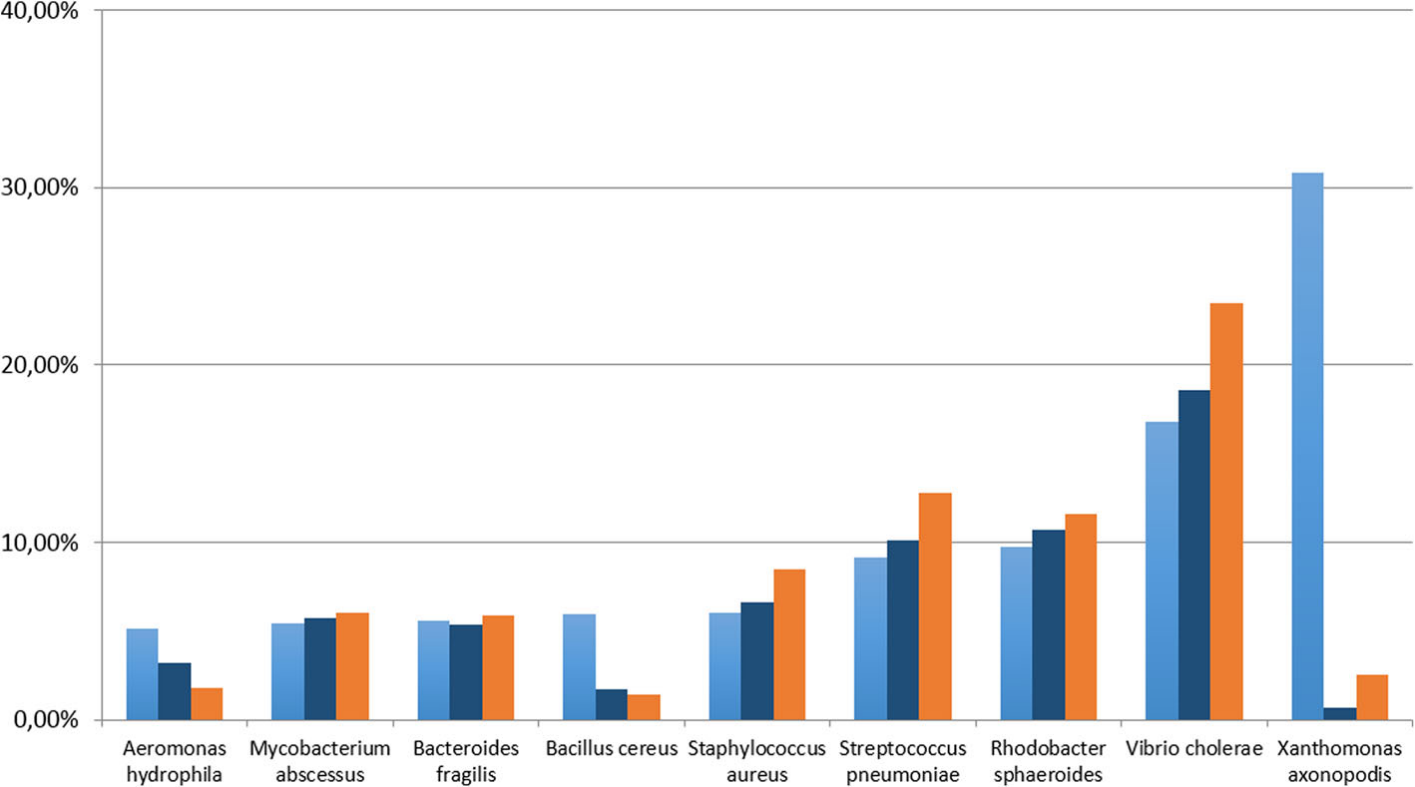
Predicted abundance at species level. Analysis on the dataset MK_a1. In light-blue the ground truth, in blue CLIOR, in orange Clark-l

In both cases the Pearson correlation coefficient for CLIOR is equal or close to *r*=1 in many cases, showing that the predicted abundances are highly correlated with the actual dataset composition. Since the result for the dataset MK_a1 showed a behavior that is not in line with the others, we further investigate this case. Figure 6 shows that, at species level, CLIOR improves the predicted abundance of most species (see Additional file 2 for more details).

**Fig. 7:**
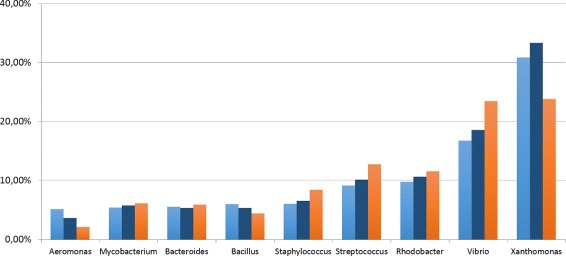
Predicted abundance at genus level. Analysis on the dataset MK_a1. In light-blue the ground truth, in blue CLIOR, in orange Clark-l

The cases of *Xanthomonas axonopodis* and *Bacillus cereus* deserve an ad-hoc discussion, as both Clark-l and CLIOR basically miss the species. The difficulty Clark-l has in the identification of these species is possibly due to the fact that, in the dictionary, several closely related species are present, thus making it difficult the call for *Xanthomonas axonopodis* and *Bacillus cereus*. In these particular cases, we speculate that CLIOR is probably mislead by the presence of these other labels, that although “close” are not correct, and extend them to other *Xanthomonas axonopodis* and *Bacillus cereus* reads. This explanation is supported by the data shown in Fig. 7 (see Additional file 2), where the abundance prediction at genus level, which will aggregate close species labels under the same genus label, is correct, and CLIOR is able to improve, with respect to Clark-l, even for genus *Xanthomonas* and *Bacillus*.

**Fig. 8:**
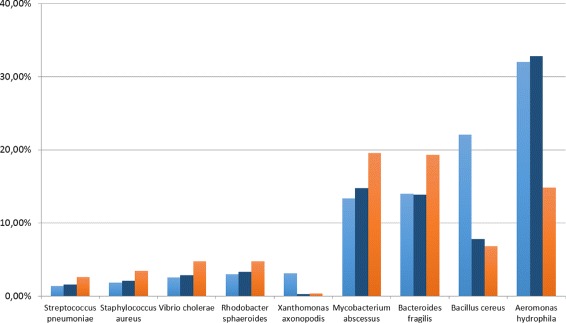
Predicted abundance at species level. Analysis on the dataset MK_a2. In light-blue the ground truth, in blue CLIOR, in orange Clark-l

**Fig. 9:**
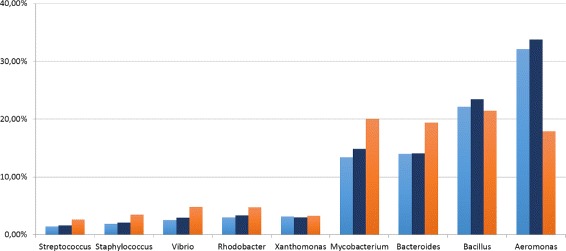
Predicted abundance at genus level. Analysis on the dataset MK_a2. In light-blue the ground truth, in blue CLIOR, in orange Clark-l

Similarly, we investigated further on the actual distribution of abundances for the dataset MK_a2, for which we have the best improvement in terms of Pearson correlation. The results are shown in Figs. 8 and 9 for species and genus level of classification, respectively.

Of particular interest is the species *Aeromonas hydrophila*, where Clark-l predicts 14.84%, CLIOR 32.80%, and the real abundance is 32.06%. The *Xanthomonas axonopodis* and *Bacillus cereus* are the most difficult species to correctly predict for both classifiers. However, similarly to what we discussed for the MK_a1 dataset, at genus level this effect disappear and the predictions are much closer to the ground truth. This indicates that several closely related species are present in the dictionary, thus affecting the labeling of Clark-l and thus of CLIOR. Overall we see that for most species the prediction made by CLIOR is actually closer to the ground truth, independently from the fact that Clark-l underestimates or overestimates the true abundance.

### Impact of the dataset size

An important feature of our approach is that the larger the read dataset in input, the better the classification. This is because the grouping phase benefits of the presence of more reads that allows in turn to better characterize the groups.

**Fig. 10:**
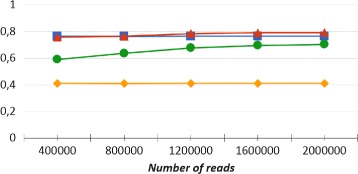
Precision and Recall as a function of the size of the input. In blue Clark-l precision, in yellow Clark-l recall. In red CLIOR precision, in green CLIOR recall

For this experiment we took subsamples of increasing size from the MK_a2 dataset, which contains 1M paired end reads (i.e. 2M reads in total). Figure 10 shows precision and recall at species level, as a function of the size of the dataset.

**Fig. 11:**
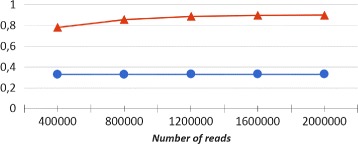
Pearson correlation as a function of the size of the input. In blue Clark-l, in red CLIOR

The same considerations holds for the Pearson correlation, as shown in Fig. 11.

**Fig. 12:**
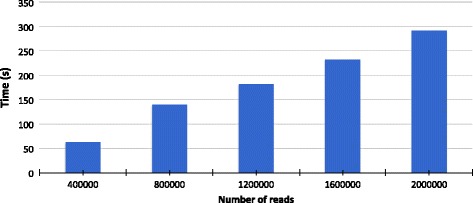
Time needed for the overall classification task. The total time to obtain the final classification. Time is shown as a function of the dataset size on subsets of increasing size for MK_a2, which contains 1M paired end reads, i.e. 2M read in total

In terms of time performances, the total time depends, obviously, on the dataset size. Figure 12 shows the total time needed to complete the classification process as a function of the input size. It can be seen how the time slightly increases, taking less than 5 min to analyse 2M reads.

The size of the dataset also affects the quality of the classification itself. In fact, the larger the number of reads available, the better is the quality of the groups created by CLIOR.

### Results on real metagenomes

We tested CLIOR also on real datasets in order to validate the performances of the classification process on real metagenomic samples for which the ground truth is not known. We used Clark-l and CLIOR to classify Human Microbiome Project reads, also used in [10, 11], in order to compare the number of assigned reads (see Table 2).

**Table 2:** Number of assigned reads on real samples: comparison between Clark-l and CLIOR

Datasets	Tool	Species	Genus
SRR062276	Clark-l	0.220	0.221
	CLIOR	0.523	0.523
SSR062301	Clark-l	0.220	0.220
	CLIOR	0.527	0.527
SSR061942	Clark-l	0.229	0.229
	CLIOR	0.469	0.469
SSR062415	Clark-l	0.170	0.170
	CLIOR	0.266	0.302
SSR062462	Clark-l	0.171	0.171
	CLIOR	0.267	0.302

From our classifications tests, we found that on the mid-vagina samples (SRR062276 and SRR062301), Clark-l classifies 22% of reads, while CLIOR classified 52.3% of reads, more than double the reads assigned by Clark-l at species and genus levels. A similar performance was observed on the anterior nares (SRR061942) datasets, where Clark-l classifies only 23% of reads and CLIOR classified twice as many reads, about 47% at both levels. For the saliva samples (SRR062415 and SRR062462), Clark-l classifies 17% both at the species and genus level, whereas CLIOR assigned 26.6% and 30.2%, respectively. In this case the increment is about 9% at species level and 13% at genus level.

**Fig. 13:**
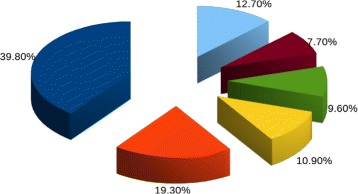
Analysis on real data. Top 5 genera detected by CLIOR in the saliva datasets SRR062415: Streptococcus (blue), Haemophilus (orange), Prevotella (yellow), Azotobacter (green), Neisseria (maroon), Others (light blue)

The species and genera found in these real samples can be investigated further by comparing the abundance ratios. For example in the saliva dataset (SRR062415) the top five genera found by CLIOR are *Streptococcus, Haemophilus, Prevotella, Azotobacter* and *Neisseria* as shown in Fig. 13. These genera have been also reported as the most abundant by [10, 11, 20], with similar ratios. Similar observations can be drawn from the other real samples (see Additional file 4). In summary, CLIOR is able to double the number of reads assigned to some species also in real datasets. This is important because the classification of more reads allows a more robust identification of species in a sample. Moreover, the resulting abundance ratios of the species are in line with previous studies.

## Conclusions

In this paper we have introduced CLIOR (CLassification Improvement with Overlapping Reads), a metagenomic classification booster that is based on the overlapping reads graph. CLIOR is not restricted to the single reads classification algorithm, e.g. Clark-l, and it may be applied to other methods such as, for example, Kraken. Results on several simulated metagenomes show that CLIOR is able to improve both the recall and the precision with respect to Clark-l: at species level, on average, the increment is 41.9% and 10.9% respectively, while at genus level it is 35.7% and 0.4% respectively. Results on more realistic synthetic metagenomes confirmed that CLIOR can improve the recall substantially (on average, 11% at species level and 15.9% at genus level) at a cost of a small loss in terms of precision (on average, 2.6% at species level, and 1% at genus level). Moreover, on real samples CLIOR was able to classify a substantially larger number of reads than Clark-l, most of the time, doubling the recall. CLIOR does not need large computational resources and it can be run on a laptop.

## Additional files

Additional file 1 Results without re-assignment (PDF file). The file contains tables showing the precision and recall at species and genus level without re-labeling for simulated and synthetic dataset, respectively. (PDF 48 kb).

Additional file 2 Details of classification for the dataset MK_a1 (PDF file) The file contains tables showing the details of the classification at species and genus level, respectively, for the dataset MK_a1. (PDF 37 kb)

Additional file 3 The file contains tables showing the detailed values of precision, recall, and f-measure, both at species and genus level, for Clark-l and Clior on simulated and synthetic datasets, respectively; and tables showing the number of reads that have been classified at species and genus level, respectively. (PDF 72 kb)

Additional file 4 This file contains figures showing the top 5 dominant species in several real samples. In SRS015072 (mid-vagina) we found that Lactobacillus is dominant, as in [11] and other studies cited by the same paper. Pseudomonas and Desulfotomaculum were detected as in [11] but we also found Azotobacter, Streptococcus [21, 22] and Mycoplasma that do not appear in [11]. In SRS019120 (saliva) we found Streptococcus, Haemophilus, Prevotella and Neisseria that appear also in [10, 11, 20] and also the Azotobacter genus as in mid-vagina datasets. In SRS023847 (anterior nares) the Propionibacterium and Staphylococcus is present as in [11], but with a different percentage (Propionibacterium from 61.5% in [11] to 46,10% in CLIOR). Mycoplasma appears in the result of SRS023847 with about the same abundance of Propionibacterium. This genus is not present in [11] and in our experiments with Clark-l is present in a small percentage (only about 0,42%). We can guess that there are some reads that overlap for this genus so they create some groups and the winner take all method allows to find them. In SRS023847 appear also Azotobacter, as in the previous datasets, and Bacillus, which do not appear in [11] but is an important pesticide and easily inhalable. (PDF 385 kb)
