# Supplementary material for: Higher recall in metagenomic sequence classification exploiting overlapping reads
Source: BMC Genomics. 2017 Dec 6;18(Suppl 10):917. doi: 10.1186/s12864-017-4273-6 (PMC5731601; doi:10.1186/s12864-017-4273-6)
Supplement: Supplementary file 1 — Results without re-assignment (PDF file). The file contains tables showing the precision and recall at species and genus level without re-labeling for simulated and synthetic dataset, respectively. (PDF 48 kb). [file 12864_2017_4273_MOESM1_ESM.pdf]

Additional file 1 - Results without re-assignment

Tables 1 and 2 show the precision and recall at species and genus level without re-labeling for simulated and synthetic dataset, respectively.

Table 1: Comparison of precision and recall at species and genus level **without** label re-assignment for simulated datasets.

Dataset	Classificator	Species			Genus		
		**prec**	**rec**	**F-m**	**prec**	**rec**	**F-m**
S1	Clark-l	0.983	0.584	0.733	0.996	0.592	0.743
	CLIOR	0.99	0.988	0.989	0.998	0.996	0.997
S2	Clark-l	0.965	0.507	0.664	0.983	0.516	0.677
	CLIOR	0.973	0.97	0.971	0.991	0.988	0.99
S3	Clark-l	0.972	0.409	0.576	0.982	0.413	0.582
	CLIOR	0.987	0.983	0.985	0.991	0.988	0.989
S4	Clark-l	0.98	0.938	0.958	0.992	0.951	0.971
	CLIOR	0.98	0.98	0.98	0.992	0.992	0.992
S5	Clark-l	0.965	0.468	0.63	0.976	0.474	0.638
	CLIOR	0.977	0.974	0.976	0.986	0.983	0.985
S6	Clark-l	0.968	0.749	0.844	0.994	0.769	0.867
	CLIOR	0.975	0.974	0.975	0.995	0.994	0.995
S7	Clark-l	0.973	0.782	0.867	0.977	0.785	0.871
	CLIOR	0.97	0.969	0.97	0.974	0.973	0.973
S8	Clark-l	0.972	0.723	0.829	0.981	0.731	0.838
	CLIOR	0.972	0.969	0.971	0.98	0.976	0.978
S9	Clark-l	0.851	0.518	0.644	0.989	0.603	0.749
	CLIOR	0.902	0.9	0.901	0.991	0.988	0.99
S10_S	Clark-l	0.848	0.471	0.605	0.974	0.541	0.696
	CLIOR	0.89	0.84	0.864	0.978	0.924	0.95
L1	Clark-l	0.798	0.435	0.563	0.99	0.542	0.7
	CLIOR	0.885	0.882	0.883	0.993	0.99	0.991
L2	Clark-l	0.753	0.448	0.562	0.993	0.593	0.743
	CLIOR	0.849	0.847	0.848	0.994	0.992	0.993
L3	Clark-l	0.733	0.456	0.562	0.994	0.62	0.764
	CLIOR	0.83	0.828	0.829	0.995	0.992	0.994
L4	Clark-l	0.721	0.459	0.561	0.995	0.635	0.775
	CLIOR	0.819	0.817	0.818	0.996	0.994	0.995
L5	Clark-l	0.714	0.462	0.561	0.995	0.646	0.784
	CLIOR	0.809	0.807	0.808	0.996	0.994	0.995
L6	Clark	0.709	0.464	0.561	0.996	0.655	0.79
	CLIOR	0.805	0.804	0.805	0.996	0.995	0.995
**Mean**	Clark-l	0.869	0.555	0.67	0.988	0.629	0.762
	CLIOR	0.913	0.908	0.911	0.99	0.985	0.988
	**Difference**	**0.044**	**0.354**	**0.241**	**0.002**	**0.356**	**0.226**

Table 2: Comparison of precision and recall at species and genus level **without** label re-assignment for synthetic datasets.

Dataset	Classificator	Species			Genus		
		**prec**	**rec**	**F-m**	**prec**	**rec**	**F-m**
HiSeq	Clark-l	0.848	0.537	0.658	0.984	0.624	0.764
	CLIOR	0.819	0.585	0.683	0.981	0.702	0.818
MiSeq	Clark-l	0.522	0.211	0.3	0.633	0.256	0.364
	CLIOR	0.467	0.345	0.397	0.548	0.405	0.466
simBA5	Clark-l	0.825	0.441	0.575	0.931	0.499	0.65
	CLIOR	0.824	0.444	0.577	0.93	0.502	0.652
MK_a1	Clark-l	0.74	0.526	0.615	0.988	0.703	0.822
	CLIOR	0.638	0.581	0.608	0.986	0.899	0.941
MK_a2	Clark-l	0.765	0.412	0.535	0.975	0.526	0.683
	CLIOR	0.758	0.672	0.712	0.977	0.866	0.919
Mean	Clark-l	0.74	0.425	0.537	0.902	0.522	0.656
	CLIOR	0.701	0.525	0.595	0.885	0.675	0.759
	**Difference**	**-0.039**	**0.1**	**0.059**	**-0.017**	**0.153**	**0.103**
