# Supplementary material for: Higher recall in metagenomic sequence classification exploiting overlapping reads
Source: BMC Genomics. 2017 Dec 6;18(Suppl 10):917. doi: 10.1186/s12864-017-4273-6 (PMC5731601; doi:10.1186/s12864-017-4273-6)
Supplement: Supplementary file 2 — Details of classification for the dataset MK_a1 (PDF file) The file contains tables showing the details of the classification at species and genus level, respectively, for the dataset MK_a1. (PDF 37 kb) [file 12864_2017_4273_MOESM2_ESM.pdf]

Additional file 2 - Details of classification for the dataset MK_a1 (PDF file)

Tables 1 and 2 show the details of the classification at species and genus level, respectively, for the dataset MK_a1.

Table 1: Details of the species-level classification for the synthetic dataset MK_a1.

	Ground Truth		Clark-l					CLIOR				
Species	reads	adund	assigned	abund	diff	correct	precision	assigned	abund	diff	correct	precision
Staphylococcus aureus	120910	6,05%	120585	8,49%	2,44%	120521	99,95%	120278	6,60%	0,55%	120018	99,78%
Bacteroides fragilis	112292	5,61%	83613	5,88%	0,27%	83604	99,99%	97200	5,33%	0,28%	97156	99,95%
Vibrio cholerae	335414	16,77%	334024	23,51%	6,74%	333970	99,98%	338076	18,55%	1,78%	335128	99,13%
Rhodobacter sphaeroides	195446	9,77%	165118	11,62%	1,85%	165077	99,98%	194762	10,69%	0,91%	194372	99,80%
Mycobacterium abscessus	108504	5,43%	85621	6,03%	0,60%	85611	99,99%	104914	5,76%	0,33%	104894	99,98%
Streptococcus pneumoniae	183064	9,15%	181258	12,76%	3,60%	181245	99,99%	184454	10,12%	0,97%	182510	98,95%
Xanthomonas axonopodis	616042	30,80%	35778	2,52%	28,28%	35775	99,99%	12958	0,71%	30,09%	12946	99,91%
Bacillus cereus	119550	5,98%	20050	1,41%	4,57%	20032	99,91%	30910	1,70%	4,28%	30864	99,85%
Aeromonas hydrophila	103532	5,18%	25786	1,81%	3,36%	25754	99,88%	58942	3,23%	1,94%	58888	99,91%
Pelosinus fermentans	105246	5,26%	N/A									
				AVG	5,75%				AVG	4,57%		

Table 2: Details of the genus-level classification for the synthetic dataset MK_a1.

	Ground Truth		Clark-l					CLIOR				
Genus	reads	adund	assigned	abund	diff	correct	precision	assigned	abund	diff	correct	precision
Staphylococcus	120910	6,05%	120597	8,47%	2,42%	120544	99,96%	120228	6,60%	0,55%	119996	99,81%
Vibrio	335414	16,77%	334571	23,49%	6,72%	334423	99,96%	337610	18,52%	1,75%	335046	99,24%
Bacteroides	112292	5,61%	84203	5,91%	0,30%	84200	100,00%	98126	5,38%	0,23%	98082	99,96%
Rhodobacter	195446	9,77%	165005	11,59%	1,81%	164986	99,99%	194322	10,66%	0,89%	194226	99,95%
Streptococcus	183064	9,15%	181852	12,77%	3,62%	181814	99,98%	184512	10,12%	0,97%	182512	98,92%
Xanthomonas	616042	30,80%	339475	23,84%	6,96%	339417	99,98%	607182	33,31%	2,51%	606060	99,82%
Bacillus	119550	5,98%	62817	4,41%	1,57%	62703	99,82%	98190	5,39%	0,59%	97966	99,77%
Mycobacterium	108504	5,43%	87551	6,15%	0,72%	87421	99,85%	106136	5,82%	0,40%	106078	99,95%
Aeromonas	103532	5,18%	31018	2,18%	3,00%	30983	99,89%	66918	3,67%	1,50%	66850	99,90%
Pelosinus	105246	5,26%	N/A									
				AVG	3,01%				AVG	1,04%		
