# Supplementary material for: Higher recall in metagenomic sequence classification exploiting overlapping reads
Source: BMC Genomics. 2017 Dec 6;18(Suppl 10):917. doi: 10.1186/s12864-017-4273-6 (PMC5731601; doi:10.1186/s12864-017-4273-6)
Supplement: Supplementary file 3 — The file contains tables showing the detailed values of precision, recall, and f-measure, both at species and genus level, for Clark-l and Clior on simulated and synthetic datasets, respectively; and tables showing the number of reads that have been classified at species and genus level, respectively. (PDF 72 kb) [file 12864_2017_4273_MOESM3_ESM.pdf]

Additional file 3 — Detailed classification results

Table 1 and Table 2 show the detailed values of precision, recall, and f-measure, both at species and genus level, for Clark-l and Clior on simulated and synthetic datasets respectively. Table 3 and Table 4 show the number of reads that have been classified at species and genus level respectively.

Table 1: Genus-level and species-level accuracy of CLIOR and Clark-l, for various simulated metagenomes.

Dataset	Classifier	Species			Genus		
		**prec**	**rec**	**F-m**	**prec**	**rec**	**F-m**
S1	Clark-l	0.983	0.584	0.733	0.996	0.592	0.743
	CLIOR	0.998	0.996	0.997	1.000	0.998	0.999
S2	Clark-l	0.965	0.507	0.664	0.983	0.516	0.677
	CLIOR	0.974	0.971	0.973	1.000	0.997	0.999
S3	Clark-l	0.972	0.409	0.576	0.982	0.413	0.582
	CLIOR	0.993	0.989	0.991	0.993	0.989	0.991
S4	Clark-l	0.980	0.938	0.959	0.992	0.951	0.971
	CLIOR	0.999	0.999	0.999	0.999	0.999	0.999
S5	Clark-l	0.965	0.468	0.630	0.976	0.474	0.638
	CLIOR	0.983	0.980	0.982	0.994	0.991	0.992
S6	Clark-l	0.968	0.749	0.844	0.994	0.769	0.867
	CLIOR	0.999	0.998	0.999	0.999	0.998	0.999
S7	Clark-l	0.973	0.782	0.867	0.977	0.785	0.871
	CLIOR	0.965	0.964	0.965	0.965	0.964	0.965
S8	Clark-l	0.972	0.724	0.829	0.981	0.731	0.838
	CLIOR	0.969	0.966	0.968	0.970	0.966	0.968
S9	Clark-l	0.852	0.518	0.644	0.989	0.603	0.749
	CLIOR	0.973	0.970	0.972	0.992	0.989	0.990
S10_S	Clark-l	0.848	0.471	0.605	0.974	0.541	0.696
	CLIOR	0.908	0.857	0.882	0.981	0.927	0.953
L1	Clark-l	0.798	0.435	0.563	0.990	0.542	0.700
	CLIOR	0.981	0.978	0.979	0.993	0.990	0.992
L2	Clark-l	0.753	0.448	0.562	0.993	0.593	0.743
	CLIOR	0.984	0.982	0.983	0.995	0.993	0.994
L3	Clark-l	0.733	0.456	0.562	0.994	0.620	0.764
	CLIOR	0.982	0.980	0.981	0.996	0.994	0.995
L4	Clark-l	0.722	0.459	0.561	0.995	0.635	0.775
	CLIOR	0.983	0.981	0.982	0.998	0.996	0.997
L5	Clark-l	0.714	0.462	0.561	0.995	0.646	0.784
	CLIOR	0.977	0.975	0.976	0.998	0.996	0.997
L6	Clark-l	0.709	0.464	0.561	0.996	0.655	0.790
	CLIOR	0.986	0.984	0.985	0.998	0.996	0.997
Mean	Clark-l	0.869	0.555	0.670	0.988	0.629	0.762
	CLIOR	0.978	0.973	0.976	0.992	0.986	0.989
	**Difference**	**0.109**	**0.419**	**0.306**	**0.004**	**0.357**	**0.227**

Table 2: Genus-level and species-level accuracy of Clark-l and CLIOR, for various synthetic metagenomes.

**Dataset**	**Classifier**	**Species**			**Genus**		
		**prec**	**rec**	**F-m**	**prec**	**rec**	**F-m**
HiSeq	Clark-l	0.848	0.537	0.658	0.984	0.624	0.764
	CLIOR	0.822	0.587	0.685	0.982	0.702	0.819
MiSeq	Clark-l	0.522	0.211	0.300	0.633	0.256	0.364
	CLIOR	0.511	0.378	0.434	0.570	0.421	0.484
simBA5	Clark-l	0.825	0.441	0.575	0.931	0.499	0.650
	CLIOR	0.822	0.443	0.576	0.928	0.500	0.650
MK_a1	Clark-l	0.740	0.526	0.615	0.988	0.703	0.822
	CLIOR	0.623	0.568	0.594	0.991	0.903	0.945
MK_a2	Clark-l	0.765	0.412	0.535	0.975	0.526	0.683
	CLIOR	0.793	0.703	0.745	0.987	0.875	0.928
Mean	Clark-l	0.740	0.425	0.537	0.902	0.522	0.656
	CLIOR	0.714	0.536	0.607	0.892	0.680	0.765
	**Difference**	**-0.026**	**0.110**	**0.070**	**-0.010**	**0.159**	**0.109**

Table 3: Number of reads classified at species-level by Clark-l and CLIOR, for various synthetic and simulated metagenomes.

Dataset	Clark-l		CLIOR		
	Correct	Classified	Correct	Classified	Total
HiSeq	5369370	6331519	5871490	7144305	10000000
MiSeq	843263	1616820	1509860	2955748	4000000
simBA5	1324270	1604999	1328590	1616134	3000000
MK_a1	1051590	1420798	1135770	1822420	2000000
MK_a2	823061	1076595	1404950	1772668	2000000
S1.fna	112642	114542	191946	192386	192734
S2.fna	197942	205175	379480	389632	390678
S3.fna	277134	284987	670172	675164	677450
S4.fna	704257	718929	749572	750420	750578
S5.fna	304505	315678	637852	648860	650800
S6.fna	1068000	1103043	1424040	1425418	1426760
S7.fna	2585490	2657460	3188410	3303540	3307100
S8.fna	660154	679466	881300	909162	912448
S9.fna	2315120	2718989	4335380	4454982	4468340
S10_S	1411850	1664369	2571620	2833176	3000000
L1.fna	153797	192719	345502	352176	353376
L2.fna	232576	308919	509760	517868	519136
L3.fna	312153	425604	671096	683370	684896
L4.fna	390346	541022	834516	849022	850656
L5.fna	469281	657274	990554	1014408	1016420
L6.fna	548592	774223	1163700	1179950	1182180

Table 4: Number of reads classified at genus-level by Clark-l and CLIOR, for various synthetic and simulated metagenomes.

Dataset	Clark-l		CLIOR		
	Correct	Classified	Correct	Classified	Total
HiSeq	6242990	6341992	7022260	7150176	10000000
MiSeq	1022350	1615226	1683190	2953717	4000000
simBA5	1496150	1606824	1500920	1617366	3000000
MK_a1	1406490	1424091	1806830	1822582	2000000
MK_a2	1051440	1078781	1750100	1772912	2000000
S1.fna	114096	114556	192384	192386	192734
S2.fna	201749	205289	389562	389634	390678
S3.fna	279908	284987	670196	675164	677450
S4.fna	713474	719353	749644	750420	750578
S5.fna	308239	315846	644862	648864	650800
S6.fna	1097280	1103946	1424140	1425428	1426760
S7.fna	2597400	2658211	3188420	3303544	3307100
S8.fna	666976	679748	881402	909164	912448
S9.fna	2695160	2725240	4417700	4455056	4468340
S10_S	1623410	1666794	2779380	2833444	3000000
L1.fna	191397	193276	349782	352178	353376
L2.fna	307842	310034	515454	517878	519136
L3.fna	424768	427322	680482	683380	684896
L4.fna	540436	543343	847094	849028	850656
L5.fna	656977	660108	1012740	1014428	1016420
L6.fna	774294	777697	1177340	1179958	1182180
