# Supplementary material for: Higher recall in metagenomic sequence classification exploiting overlapping reads
Source: BMC Genomics. 2017 Dec 6;18(Suppl 10):917. doi: 10.1186/s12864-017-4273-6 (PMC5731601; doi:10.1186/s12864-017-4273-6)
Supplement: Supplementary file 4 — This file contains figures showing the top 5 dominant species in several real samples. In SRS015072 (mid-vagina) we found that Lactobacillus is dominant, as in [11] and other studies cited by the same paper. Pseudomonas and Desulfotomaculum were detected as in [11] but we also found Azotobacter, Streptococcus [21, 22] and Mycoplasma that do not appear in [11]. In SRS019120 (saliva) we found Streptococcus, Haemophilus, Prevotella and Neisseria that appear also in [10, 11, 20] and also the Azotobacter genus as in mid-vagina datasets. In SRS023847 (anterior nares) the Propionibacterium and Staphylococcus is present as in [11], but with a different percentage (Propionibacterium from 61.5% in [11] to 46,10% in CLIOR). Mycoplasma appears in the result of SRS023847 with about the same abundance of Propionibacterium. This genus is not present in [11] and in our experiments with Clark-l is present in a small percentage (only about 0,42%). We can guess that there are some reads that overlap for this genus so they create some groups and the winner take all method allows to find them. In SRS023847 appear also Azotobacter, as in the previous datasets, and Bacillus, which do not appear in [11] but is an important pesticide and easily inhalable. (PDF 385 kb) [file 12864_2017_4273_MOESM4_ESM.pdf]

Additional file 4 — Detailed classification results

Figures 1, to 5 shows the top 5 dominant species in several real samples.

In SRS015072 (mid-vagina) we found that Lactobacillus is dominant, as in [1] and other studies cited by the same paper. Pseudomonas and Desulfotomaculum were detected as in [1] but we also found Azotobacter, Streptococcus [2, 3] and Mycoplasma that do not appear in [1]. In SRS019120 (saliva) we found Streptococcus, Haemophilus, Prevotella and Neisseria that appear also in [1, 4, 5] and also the Azotobacter genus as in mid-vagina datasets. In SRS023847 (anterior nares) the Propionibacterium and Staphylococcus is present as in [1], but with a different percentage (Propionibacterium from 61.5% in [1] to 46,10% in CLIOR). Mycoplasma appears in the result of SRS023847 with about the same abundance of Propionibacterium. This genus is not present in [1] and in our experiments with Clark-l is present in a small percentage (only about 0,42%). We can guess that there are some reads that overlap for this genus so they create some groups and the winner take all method allows to find them. In SRS023847 appear also Azotobacter, as in the previous datasets, and Bacillus, which do not appear in [1] but is an important pesticide and easily inhalable.

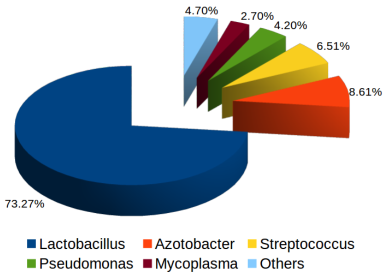

Figure 1: Top 5 genera of classified reads in mid-vagina SRR062276 dataset.

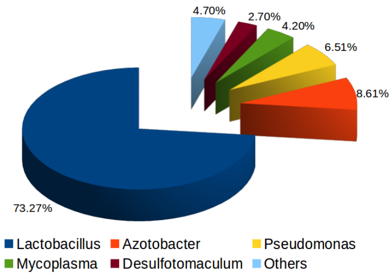

Figure 2: Top 5 genera of classified reads in mid-vagina SRR062301 dataset.

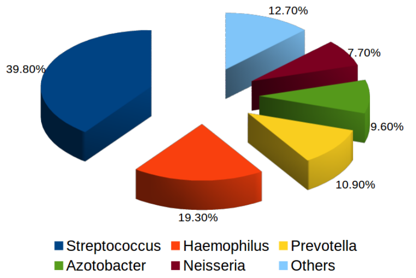

Figure 3: Top 5 genera detected by CLIOR in the saliva SRR062415 dataset.

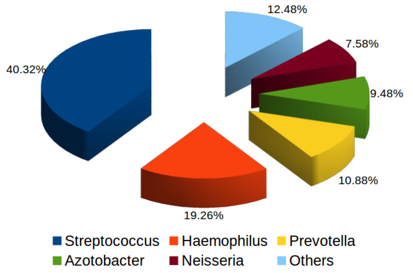

Figure 4: Top 5 genera of classified reads in the saliva SRR062462 dataset.

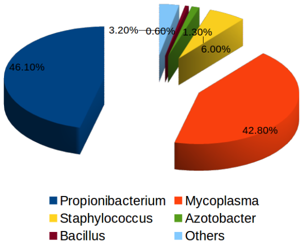

Figure 5: Top 5 genera of classified reads in anterior nares SRR061942 dataset.

References

[1] Ounit, R., Wanamaker, S., Close, T.J., Lonardi, S.: Clark: fast and accurate classification of metagenomic and genomic sequences using discriminative k-mers. BMC Genomics **16**(1), 1–13 (2015). doi:10.1186/s12864-015-1419-2

[2] Dechen, T.C., Sumit, K., Ranabir, P.: Correlates of Vaginal Colonization with Group B Streptococci among Pregnant Women. Journal of Global Infectious Diseases **2**(3), 236–241 (2010). doi:10.4103/0974-777X.68536. Accessed 2016-06-07TZ

[3] Verstraelen, H., Verhelst, R., Vaneechoutte, M., Temmerman, M.: Group A streptococcal vaginitis: an unrecognized cause of vaginal symptoms in adult women. Archives of Gynecology and Obstetrics **284**(1), 95–98 (2011). doi:10.1007/s00404-011-1861-6

[4] Said, H.S., Suda, W., Nakagome, S., Chinen, H., Oshima, K., Kim, S., Kimura, R., Iraha, A., Ishida, H., Fujita, J., Mano, S., Morita, H., Dohi, T., Oota, H., Hattori, M.: Dysbiosis of Salivary Microbiota in Inflammatory Bowel Disease and Its Association With Oral Immunological Biomarkers. DNA Research: An International Journal for Rapid Publication of Reports

on Genes and Genomes **21**(1), 15–25 (2014). doi:10.1093/dnares/dst037. Accessed 2016-06-07TZ

[5] Wood, D., Salzberg, S.: Kraken: ultrafast metagenomic sequence classification using exact alignments. Genome Biol. **15** (2014). doi:10.1186/gb-2014-15-3-r46
